# Cell Culture Models for Translational Research on Thymomas and Thymic Carcinomas: Current Status and Future Perspectives

**DOI:** 10.3390/cancers16152762

**Published:** 2024-08-04

**Authors:** Denise Müller, Jürgen Loskutov, Stefan Küffer, Alexander Marx, Christian R. A. Regenbrecht, Philipp Ströbel, Manuela J. Regenbrecht

**Affiliations:** 1 Institute of Pathology, University Medical Center Göttingen, 37075 Göttingen, Germany; stefan.kueffer@med.uni-goettingen.de (S.K.); christian.regenbrecht@asc-oncology.com (C.R.A.R.);; 2 CELLphenomics GmbH, 13125 Berlin, Germanymanuela.regenbrecht@asc-oncology.com (M.J.R.); 3 ASC Oncology GmbH, 13125 Berlin, Germany; 4 Department for Pneumology, Palliative Medicine, DRK Kliniken Berlin, 14050 Berlin, Germany

**Keywords:** thymic epithelial tumors, patient-derived cell culture, molecular mechanisms, targeted therapies, personalized medicine

## Abstract

**Simple Summary:**

Thymomas and thymic carcinomas (together referred to as thymic epithelial tumors, TETs) are a heterogeneous group of rare tumors of the anterior mediastinum. Most treatment regimens for advanced TETs are empirical and patients have not benefited from developments in personalized medicine. We believe that one of the major unresolved problems in TET research is the lack of suitable cellular or animal models that represent the full heterogeneity of TETs, help us to study the driving mechanisms behind TET biology, and explore vulnerabilities. In this narrative review, we discuss the current state of 2D and 3D TET cell culture models and protocols. To move beyond the current state, innovative high-throughput techniques are mandatory for both systematic optimization of cell culture conditions and high-throughput drug screening.

**Abstract:**

Cell culture model systems are fundamental tools for studying cancer biology and identifying therapeutic vulnerabilities in a controlled environment. TET cells are notoriously difficult to culture, with only a few permanent cell lines available. The optimal conditions and requirements for the ex vivo establishment and permanent expansion of TET cells have not been systematically studied, and it is currently unknown whether different TET subtypes require different culture conditions or specific supplements. The few permanent cell lines available represent only type AB thymomas and thymic carcinomas, while attempts to propagate tumor cells derived from type B thymomas so far have been frustrated. It is conceivable that epithelial cells in type B thymomas are critically dependent on their interaction with immature T cells or their three-dimensional scaffold. Extensive studies leading to validated cell culture protocols would be highly desirable and a major advance in the field. Alternative methods such as tumor cell organoid models, patient-derived xenografts, or tissue slices have been sporadically used in TETs, but their specific contributions and advantages remain to be shown.

## 1. Introduction

Thymic epithelial tumors (TETs) are heterogeneous neoplasms arising from the epithelial cells of the thymus. They are classified into thymomas and thymic carcinomas, based on differences in biology, morphology, and clinical behavior [1]. Although they represent the most common malignancies of the anterior mediastinum, TETs account for only 0.2% to 1.5% of all adult cancers, with an annual incidence of 1.3 cases per million people [2]. Thymomas are 10 times more common than thymic carcinomas. Thymomas are further subdivided into five major groups ranging from highly organoid tumors with a high content of immature T cells that resemble a normal thymus and recapitulate critical thymic functions to aggressive tumors with the capacity to metastasize. Thymic carcinomas, on the other hand, are invariably aggressive tumors with local invasion of adjacent structures and distant metastasis and generally have a poor prognosis [3]. TETs may remain asymptomatic for a long time or may present with chest pain, cough, or dyspnea. Approximately 30–50% of thymomas come to clinical attention because of their association with myasthenia gravis, an autoimmune disorder that affects the neuromuscular junction of striated muscle fibers [4]. Compared to other cancers in the body, the prognosis of most localized thymomas is favorable, with 5-year survival rates between 70% and 95% [5,6,7]. However, patients with advanced or recurrent disease require multimodal treatment combining surgery, chemotherapy, and radiation [8,9,10] and face a poor prognosis, with 5-year survival rates of 20% [6]. Second-line therapies are largely empirical and currently not standardized [11,12,13]. Thus, there is an urgent medical need to improve the treatment of TET patients through systematic translational research. However, almost all of the published scientific data on TETs over the past few decades have been based on studies of fresh frozen, or paraffin-embedded tissue samples with inherent methodological limitations, with the consequence that we still know very little about the functional properties of TET cells in vivo. TETs are one of the adult cancers with the lowest mutational burden [14], with no known targetable oncogenic drivers so far [14,15]. The fact that the molecular factors driving TET development are still poorly understood is also the reason why there are currently no endogenous animal models, with the important exception of recent mouse models with *GTF2I* mutations [16,17]. Patient-derived xenograft mouse models could be a valuable alternative and have been successfully used in TETs [18]. Cell culture models are at the core of any translational study and provide an irreplaceable platform to study the functional consequences of genetic alterations and dysregulated signaling pathways in a controlled environment. New model systems and advanced techniques to establish and study living TET cells will be essential to finally unravel TET biology and to improve the clinical outcome of patients with advanced or recurrent TETs.

## 2. Permanent 2D TET Cell Lines

In spite of many attempts by several groups worldwide, only four permanent thymic carcinoma and three thymoma cell lines have been published [19] (Table 1). Which factors exactly determined the successful long-term establishment of these seven cell lines is unknown. Looking at the published permanent cell lines, it is noteworthy that all of them were derived from either type AB thymomas or thymic carcinomas. It is currently unknown whether this apparent bias is related to a specific cell population in the primary tumors and whether different thymoma subtypes and thymic carcinomas require specific growth supplements or matrices in their cell culture dish. There are no publications comparing different cell isolation or cultivation protocols.

**Figure 1:**
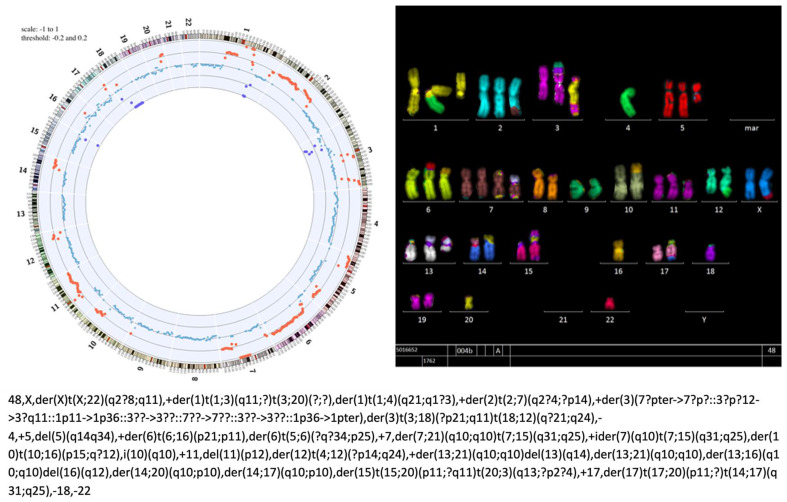
Shallow whole-genome sequencing and multi-color FISH analysis of T1889 cells showing a highly aberrant karyotype (courtesy of Dr. Katayoon Shirneshan, University Medical Center Göttingen). Multi-color FISH can be used to trace the exchange of large portions of genomic material across different chromosomes (for example, note the translocation of large parts of the long arm of chr. 4 (bright green) to one of the two chromosomes 1).

The first report on a permanent thymic carcinoma cell line (Ty-82) was published in 1992 [20]. However, the authors already described a translocation t(15;19) which was later shown to lead to *BRD4-NUT* gene fusions [21], and such tumors are now classified not as thymic carcinomas but as the molecularly defined group of “NUT carcinomas of the thorax” [22]. The next reports on the successful establishment of two carcinoma cell lines came almost two decades later, in 2008, in two independent studies [23,24]. The thymic carcinoma cell line ThyL-6 was described to express multiple cytokeratins as well as EMA and CD5. The cells were shown to secrete multiple inflammatory cytokines and cell proliferation was dependent on autocrine IL-6 stimulation [23], but it was not stated whether the cells survived in the long term. The second thymic carcinoma cell line published in 2008, T1889, was derived from a poorly differentiated thymic carcinoma [24] and was later shown to carry a c.738G>T, p.M246I *TP53* mutation [25]. T1889 cells had an epithelial-like growth pattern and survived for more than 80 passages. Immunocytochemical analysis confirmed the expression of epithelial markers. Therefore, T1889c can be considered the first thymic carcinoma cell line suitable for long-term in vitro studies. It should be noted, though, that these permanent T1889 cells show multiple chromosomal alterations and are highly aberrant on multi-color FISH analyses, a feature that is usually not observed in whole-tissue extracts of thymic carcinomas (Figure 1). However, most of these aberrations were already present in the original description of this cell line [24] and are thus not a result of long-term cultivation. Still, T1889 cells have been used in several published studies and have significantly advanced the understanding of thymic carcinoma biology. The prominent anti-apoptotic signature of T1889 was important for the study of apoptotic cell death mechanisms in TETs, including the identification of potential biomarkers and therapeutic targets [26,27].

From 2008 until today, four additional TET cell lines have been published, but long-term survival above passage 80 was reported for only two of them. The type AB thymoma cell line Thy0517 had been established from a patient with type AB thymoma and paraneoplastic myasthenia gravis [28]. Thy0517 has been used in functional studies focused on biomarker discovery and immune-related abnormalities in patients with MG-associated thymomas [29,30]. T68 is another TET cell line derived from an advanced type AB thymoma. The cultured tumor cells expressed cytokeratins and were tumorigenic in a xenograft transplantation model in athymic mice [31]. Finally, the thymic carcinoma cell line MP57 was successfully established in 2016 [32]. The cultured tumor cells expressed cytokeratins, p63, and CD117/KIT and were tumorigenic in nude mice. Next-generation sequencing revealed a mutation of *PIK3R2*, offering the opportunity to analyze the efficacy of PI3K inhibitors as potential targeted therapy for a subgroup of thymoma patients with strong activation of the PI3K/AKT/mTOR pathway [32,33].

**Table 1:** Published TET cell lines.

Cell Line	Primary Tissue	Donor	Passages Survived *	Reference
Ty-82	NUT carcinoma	22-year-old male	n.s.	[20]
ThyL-6	Thymic carcinoma, undifferentiated	57-year-old male	n.s.	[23]
T1889	Thymic carcinoma, poorly differentiated	56-year-old male	85	[24]
MP57	Thymic carcinoma	45-year-old male	>80	[32]
IU-TAB-1	Thymoma, type AB	53-year-old male	n.s.	[34]
Thy0517	Thymoma, type AB	50-year-old male	160	[28]
T68	Thymoma, type AB	44-year-old male	20	[31]

n.s. = not stated; * passage numbers refer to the time of publication.

## 3. Ex Vivo Isolation and Cultivation of TET Cells

The culture of patient-derived cells is a cornerstone of biomedical research and an important tool for functional studies. However, for unknown reasons, the establishment of patient-derived TET cell culture models has proven difficult. Tissue size and quality, as well as the time between surgery (warm and cold ischemia) and processing critically determine the chances of obtaining enough viable cells for subsequent cultivation. A typical isolation protocol involves the dissection of the tissue sample into small pieces followed by several rounds of enzymatic digestion. Collagenase A, collagenase D, and liberase have been reported to be suitable enzymes for the processing of human thymic tissue, resulting in high cell yields [35,36]. Purification techniques such as density gradient centrifugation or cell sorting can be used to separate whole TET tissue cell suspensions into distinct cell populations [36,37]. Cell sorting using antibodies against EpCAM, MHC class II, or Ly51 has been used for the specific enrichment of thymic (or thymoma-derived) epithelial cells (TECs) and serves as the first step in cell characterization at the same time [37]. However, when using anti-MHC antibodies, it should be kept in mind that TECs in many thymomas show a reduced or absent expression of MHC class II molecules [38,39,40].

## 4. TEC-Tailored Media Supplements

Once isolated, the long-term culture of primary TET cells is critically dependent on optimal culture conditions. Culture media tailored to the needs of TECs are essential for sustained survival and growth and, ideally, the preservationof the molecular and phenotypic characteristics of the tissue of origin. However, the optimal composition of cell culture media and the necessary supplements have not been studied and experimentally validated in detail. Several studies have highlighted important molecular pathways, cytokines, and growth factors for the development, maintenance, and regeneration of thymic epithelial cells (Table 2). WNT4-driven activation of β-catenin-dependent canonical Wnt signaling was found to be essential for thymic epithelium maintenance. Aggressive TETs also show increased expression and secretion of WNT4 [41,42], and decreasing WNT4 levels have been associated with age-related thymic involution [42]. The short-term culture of primary TECs resulted in a complete loss of WNT4 expression and secretion [41], suggesting that WNT4 supplementation is necessary to maintain the growth of TET cells in vitro. The growth-stimulating effect of WNT4 on TECs has been attributed to the observation that FOXN1, a key regulator of TEC development and function, is a downstream target of WNT4-mediated canonical Wnt signaling [41]. Similarly, BMP4 produced by thymic endothelial cells was found to promote tissue regeneration in vivo through the positive regulation of FOXN1 expression in TECs. Furthermore, in vitro treatment of immortalized FOXN1-deficient TECs with BMP4 resulted in the re-induction of FOXN1 expression [43]. RANKL has been reported to regulate TEC homeostasis through the activation of NFkB signaling, resulting in an increased expression of cell adhesion and pro-survival genes [44]. RANKL exhibited a stimulatory effect on TEC proliferation and promoted thymic recovery in vivo and ex vivo [45,46]. In addition, several well-described growth factors such as EGF, FGF7 and FGF10, HGF, and IGF1 were found to contribute to TEC survival and proliferation [47,48,49,50,51,52,53]. Inflammatory cytokines showed both positive and negative effects on thymic epithelial maintenance. While IL1, IL7, and IL22 promote the survival and proliferation of TECs [51,54,55], IL6 and TGFβ contribute to cellular senescence and decrease the number of TECs [54,56,57]. Notably, IL1 administration in vitro was accompanied by morphological changes in TEC cells, resulting in a fibroblast-like morphology [55].

## 5. Long-Term Cultivation: Replicative Senescence and Morphological Shifts

**Figure 2:**
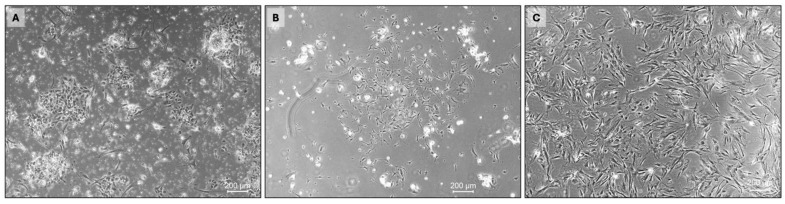
Patient-derived primary cell culture of type AB thymoma. (**A**) Whole-tissue single-cell suspensions 4 days after isolation show varying cell morphologies. (**B**) Elongated spindle cells prevail after 3 weeks and thereafter (**C**).

Ideally, primary cell culture models should mirror the morphological, phenotypic, and genotypic characteristics of their tissue of origin and capture the complexity of TET biology. Two major problems identified in previous studies were the lack of expression of important TET cell markers and the lack of similarity to the primary tissue. Immunohistochemistry, genetic profiling, and multi-omics studies have been used to characterize newly established primary cell models. However, the disruption of the tissue microenvironment and exposure of the cells to laboratory culture conditions exert selective pressure that can lead to substantial phenotypic and genotypic changes [62]. In particular, cell lines established from late-stage cancers are prone to accumulate genetic alterations during repeated passages and long-term in vitro cultivation [63] (Figure 2). In addition, repeated passaging often leads to telomere shortening and replicative senescence and limits the proliferation of human cells. Immortalization strategies such as the ectopic expression of telomerase reverse transcriptase (TERT) to reinduce telomerase activity or transformation with viral oncoproteins (e.g., SV40 large T antigen) can be used to establish stable cell lines with an unlimited proliferation capacity. Increased telomerase activity has been observed in TETs and correlated with the clinical stage of thymic carcinoma, suggesting the presence of immortalized cells in late-stage disease [64,65]. The immortalization of mouse thymic epithelial cells by retroviral transduction with SV40 large T antigen has been reported previously. Immortalized mouse TECs retained the morphological and biological properties of the original primary cells [66]. In fact, even metabolic pathways were maintained during immortalization, although the immortalized mouse TECs expressed unique glycolipid profiles [67]. Another approach that has not been tried to the best of our knowledge could be the reprogramming of TET-derived tumor cells into induced pluripotent stem cells (iPSCs) [68].

TETs are characterized by a complex microenvironment that allows epithelial tumor cells to interact with stromal cells, immune cells, and the extracellular matrix. These interactions are only incompletely understood in TET but may have a significant impact on their biology and consequently their clinical behavior and response to therapy. Indeed, whole-tissue single-cell suspensions obtained by enzymatic digestion of TET tissue samples mainly contain stromal cells and lymphocytes, with only a small fraction of epithelial tumor cells [36]. The cultivation of such unsorted cell suspensions results in a mixed culture characterized by widely varying cell morphologies and growth patterns four days after isolation (Figure 2A). In addition to some elongated spindle cells, small islands of densely packed round cells can be observed. We empirically used a standard cell culture medium (RPMI1640), supplemented with fetal calf serum, penicillin/streptomycin, and L-glutamine, which was changed every 3–4 days. Regular cold trypsinization was performed to remove fibroblasts. Different cell morphologies suggesting a mixture of stromal cells and epithelial tumor cells were maintained even after three weeks in culture (Figure 2B). An increasing proportion of elongated spindle cells over time indicated that the applied culture conditions favored the growth and survival of a specific cell type (Figure 2C). The initial observations suggested a lack of essential nutrients, growth factors, and cytokines, but also raised the question of whether the cultivation as a monolayer on a flat surface could be a surrogate for the three-dimensional TEC network and complex microenvironment of TETs.

## 6. Advanced 3D TET Cell Culture Models

**Figure 3:**
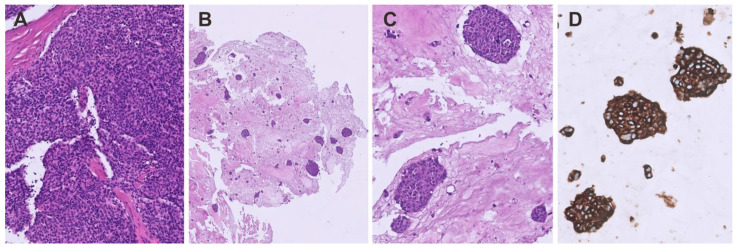
Representative images of a matrix-embedded, patient-derived 3D thymoma organoid model. (**A**) Original tumor (HE, ×200), (**B**) Tumor-derived organoids (overview, HE, ×100) (**C**) detail of (**B**) (HE, ×250), (**D**) keratin 19 staining (immunohistochemistry, ×250).

Three-dimensional model systems recapitulating tumor architecture, cell–cell contacts, and cellular interactions are an increasingly recognized tool for preclinical research, and their potential for high-throughput drug testing and its predictive value is now well established across various solid tumors, including rare cancers such as sarcomas [69]. Multiple studies have shown that while the positive predictive value of whether a substance effective in vitro is also effective in the clinical setting is around 80%, the negative predictive value is almost 100% [70,71,72]. Despite the wide use of 3D organoids, TETs are lagging behind. The first report on the generation of functional thymic organoids from human pluripotent stem cells was published in 2023 [73]. The establishment of TEC organoids from mouse thymic tissues suitable for long-term cultivation has also been described [74]. Based on a previously published method [75], we have now created several 3D organoid models from different thymomas in a matrix-embedded 3D organoid culture. These organoids retained many morphological and immunophenotypic features of the donor tissue even after passaging (Figure 3). However, more systematic studies are needed to determine whether these models reflect TET biology better than 2D cultures.

Despite the many advantages of 3D organoid cultures, the lack of non-epithelial components in the culture system is a major limitation. A more sophisticated approach using 3D scaffolds offers a promising strategy to imitate the thymic extracellular matrix in 3D thymic co-culture models. Artificially generated matrices consisting of fibrin gel mixed with fibroblasts have been reported to promote the proliferation, differentiation, and functionality of TECs [76]. In a later study, a 3D scaffold was directly generated from decellularized thymic tissues and proven suitable for the long-term in vitro cultivation of TECs with functional thymopoiesis [77].

## 7. Alternatives to Isolated Cell Cultures

Patient-derived xenografts (PDXs) in immunodeficient mice are an important experimental tool and have successfully been used both with thymoma-derived cell lines [34] and primary tumor tissues [18]. Tissue slices are three-dimensional tissue explants cut on a vibratome [78] that can be cultured ex vivo for various purposes. These tissue slices retain the anatomical architecture of the organ, and the cells are in their native environment. Tissue slices are believed to maintain the metabolic activity and tissue homeostasis of the tissue of origin for a limited period of time, typically several days. Tissue slices have also been prepared from a normal thymus and have been used to study highly sensitive biological processes such as negative T-cell selection [79]. To the best of our knowledge, this very promising technique has so far not been used or published on TETs.

## 8. Future Directions

**Figure 4:**
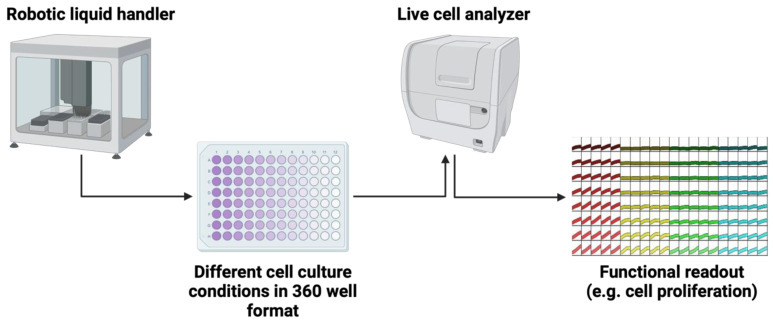
Automated workflow for optimization of comprehensive drug testing of TET cells (created with https://BioRender.com).

The establishment of functional cell culture models is clearly an unachieved but eminently important goal for translational research on TETs. It will also be important to define protocols to compare primary tumors and derived cellular models to better understand selection biases introduced by different cell culture methods. Due to the rarity of these tumors even in large, specialized centers, the establishment of such models must be systematic and cannot rely on “trial-and-error” approaches. Modern live-cell imaging techniques allow for the simultaneous cultivation and observation of a large number of living cells. By systematically testing different additives, growth factors, and culture media, this technique can be used, e.g., for establishing optimal culture conditions, systematic drug testing, or CRISPR/CAS9 knockout screens in TETs (Figure 4). Long-term experiments do not necessarily have to be the main focus here: short-term incubations to evaluate possible targeted therapies, e.g., as part of a molecular tumor board, are also conceivable and have been previously reported [25,80].

## 9. Concluding Remarks

Despite the rarity of thymic epithelial tumors (TETs), there is an urgent clinical need for novel and innovative therapeutic options to enhance outcomes, particularly for patients with late-stage and advanced disease. Cell culture model systems have become fundamental tools for studying cancer pathophysiology, exploring tumor heterogeneity, investigating molecular mechanisms, and identifying therapeutic vulnerabilities within a controlled environment. However, in the context of TETs, the absence of robust cell culture models significantly impedes these research efforts. While cell lines such as T1889, Thy0517, and MP57 have significantly advanced our understanding of TET biology, they are insufficient to capture the full heterogeneity and clinical diversity of TETs. The establishment of long-term TET cell culture models that accurately reflect the disease’s diversity is a critical challenge that must be addressed to advance TET research and improve clinical outcomes for patients.

## Figures and Tables

**Table 2:** Growth factors and cytokines with regulatory effects on TECs.

Molecule	TEC-Related Function	Reference
BMP4	- Stimulates the expression of FOXN1 in TECs - Promotes thymic recovery after damage - Mainly produced by thymic endothelial cells	[43]
CD40L	- Contributes to the development and maintenance of TECs	[58]
EGF	- Supports the proliferation and maintenance of TECs	[49]
FGF7/KGF	- Promotes TEC proliferation but not differentiation - Has a positive effect on thymic regeneration - Protects the thymic microenvironment to promote thymopoiesis	[47,51]
FGF10	- Promotes TEC proliferation but not differentiation	[48]
HGF	- Contributes to the development and maintenance of TECs - Promotes the proliferation and survival of TECs - Supports the development and maturation of T cells	[52,53]
IGF1	- Has a positive effect on TEC proliferation and survival - Facilitates TEC–thymocyte adhesion	[50]
IL1	- Induces DNA synthesis and stimulates TEC proliferation - IL1-treated TECs present with a fibroblast-like morphology - IFNy and TNFa enhance the effect of IL1	[55]
IL6	- Mediates EMT in primary TECs - Promotes cellular senescence	[54]
IL7	- Produced by TECs - Essential for the proliferation, survival, and maturation of thymocytes	[59]
IL22	- Has a positive effect on thymic recovery after injury	[51]
RANKL	- Contributes to the development and maintenance of TECs - Dose- and time-dependent stimulation of TEC proliferation - Activates thymic recovery in vivo and ex vivo - Regulates the expression of cell adhesion (ICAM-1 and VCAM-1) and survival (BCL-2, BCL-xL, Bax) genes - Has a positive effect on the adhesion of thymocytes to TECs, facilitating T-cell differentiation	[46,51,58,60]
TGFb	- Negative regulator of TEC number	[56,57]
WNT4	- Positive correlation with the expression of FOXN1 - Critical for the maintenance of the thymic epithelium - Declining Wnt4 levels contribute to TEC senescence and thymic atrophy	[41,42,61]
